# Study of the Effects of Monacolin K and Other Constituents of Red Yeast Rice on Obesity, Insulin-Resistance, Hyperlipidemia, and Nonalcoholic Steatohepatitis Using a Mouse Model of Metabolic Syndrome

**DOI:** 10.1155/2012/892697

**Published:** 2012-12-20

**Authors:** Makoto Fujimoto, Koichi Tsuneyama, Shao-Yuan Chen, Takeshi Nishida, Jiun-Liang Chen, Yen-Chen Chen, Takako Fujimoto, Johji Imura, Yutaka Shimada

**Affiliations:** ^1^Department of Japanese Oriental Medicine, Graduate School of Medicine and Pharmaceutical Sciences, University of Toyama, Toyama 930-0194, Japan; ^2^Department of Diagnostic Pathology, Graduate School of Medicine and Pharmaceutical Sciences, University of Toyama, Toyama 930-0194, Japan; ^3^School of Medicine, Fu Jen Catholic University, New Taipei City 24205, Taiwan; ^4^Department of Hyperbaric Medicine, Cardinal Tien Hospital, Hsintien, Taipei County 231, Taiwan; ^5^Department of Traditional Chinese Medicine, Chang Gung Memorial Hospital and Chang Gung University, Taoyuan 333, Taiwan; ^6^Graduate Institute of Life Sciences, National Defense Medical Center, Taipei 114, Taiwan; ^7^Department of Environment and Humanity, Faculty of Human Development, University of Toyama, Toyama 930-8555, Japan

## Abstract

*Purpose*. Nonalcoholic fatty liver disease (NAFLD) is a progressive and intractable disease associated with metabolic syndrome. Red yeast rice (RYR) contains monacolin K, a potent inhibitor of HMG-CoA reductase, and its consumption decreases cholesterol and triglyceride levels. We examined the efficacy of RYR constituents using a novel metabolic syndrome-NAFLD mouse model (MSG mice). *Methods*. Two types of RYR grown under different culture conditions were used. 1P-DU contained only 0.002 g/100 g of monacolin K, whereas 3P-D1 contained 0.131 g/100 g. MSG mice were divided into three groups: control (C) group fed standard food, RYR-C group fed standard food with 1% 1P-DU, and RYR-M group fed standard food with 1% 3P-D1. Mice were examined from 12 to 24 weeks of age. *Results*. Serum insulin, leptin, and liver damage as well as macrophage aggregation in visceral fat in RYR-C and RYR-M groups were lower than those in C group. The serum adiponectin levels in RYR-C group were significantly higher than those in RYR-M and C groups. *Conclusions*. RYR was effective against obesity-related inflammation, insulin resistance, and NAFLD in MSG mice irrespective of monacolin K levels. GABA and various peptides produced during fermentation were determined as the active constituents of RYR.

## 1. Introduction

Nonalcoholic fatty liver disease (NAFLD) is a phenotype of the metabolic syndrome in the liver. In Europe, the United States, and Asia, the number of NAFLD and obesity cases is increasing. Approximately 31% of NAFLD cases progress to nonalcoholic steatohepatitis (NASH), which is a severe form of NAFLD [1]. Furthermore, approximately 20% of NASH cases progress to liver cirrhosis after 10 years [2]. Histopathological evaluation of the liver is necessary for diagnosis of NASH, and no noninvasive diagnostic procedure is currently available. Although weight loss through lifestyle changes such as regular exercise and diet is considered as the main treatment strategy for NAFLD/NASH, it has been shown that 50% or more of NAFLD/NASH cases could not enhance and maintain an improved lifestyle [3]. Recently, the drugs pioglitazone and candesartan have been suggested for treatment of NAFLD/NASH [4, 5]; however no therapeutic drug has currently been established. 

Red yeast rice (RYR) is a widely available dietary supplement that has been used for centuries as a herbal medication in Asian countries such as China and Taiwan. It is a fermented product of rice and red yeast (*Monascus purpureus*) and has been used by the Chinese for many centuries to make rice wine, as a food preservative to maintain the taste and color of meat and fish, and for its medicinal properties. A complete and detailed description of its manufacture is found in the ancient Chinese pharmacopoeia, Bencao Gangmu, published during the Ming Dynasty (1368–1644). In this text, RYR is characterized as mild and a useful agent for improving blood circulation. In the 1970s, monacolin K, a metabolite of *Monascus* sp., was identified and shown to significantly lower serum cholesterol levels by the Japanese scholar Professor Endo [6, 7]. Monacolin K is a potent inhibitor of HMG-CoA reductase and is known as mevinolin or lovastatin (Mevacor, a drug produced by Merck & Co., Inc.) [6, 8], a popular drug worldwide [9].

Although hypercholesterolemia is often observed in patients with NAFLD, the effect of RYR on NAFLD has not been reported. Moreover, although RYR constituents vary due to changes in culture conditions during manufacturing, no study has evaluated the simultaneous effect of two or more RYR when culture conditions are altered. The aim of this study was to examine the effect of RYR on liver pathology, serum lipid, glucose, and adipocytokine levels in a novel NAFLD/NASH mouse model [10].

## 2. Materials and Methods

### 2.1. Red Yeast Rice

The two types of red yeast rice (1P-DU and 3P-D1) were kindly provided by Gunze Ltd. (Ayabe, Japan). RYR used in this study was derived from polished white rice immersed in water for 90 min, and the water was drained out later. Sterilization of rice was performed under steam pressure at 125°C for 30 min, followed by inoculation with *M. purpureus.* The rice was cultivated for 10 days between 25 and 30°C to obtain RYR. RYR was then heated for 30 min at 110°C to deactivate *M. purpureus* and enzymes. After drying to approximately 10% of its moisture content, RYR was ground and ready for use. The only difference in the manufacturing of the two types of RYR was the culture conditions. The composition of each type of RYR is shown in Table 1. Monacolin K levels were 0.002 g/100 g in 1P-DU and 0.131 g/100 g in 3P-D1. 

### 2.2. Animal Model

Twenty-seven 11-week-old male mice that were administered monosodium glutamate (MSG) at birth were purchased from the Institute for Animal Reproduction (Kasumigaura, Japan), kept in individual cages at room temperature (23 ± 1°C) under a 12 h dark/light cycle, and allowed free access to drinking water. Weight was measured after 1 week of adaptation to feeding with standard feed (CE-2, CLEA Japan, Inc., Tokyo). The mice were divided into three groups at random (9 mice/group), and experimental feeding was initiated for 12 weeks. The control group (C group) was fed CE-2, the RYR-C group was fed CE-2 and 1% 1P-DU (0.002 g/100 g monacolin K), and RYR-monacolin K (RYR-M) group was fed CE-2 and 1% 3P-D1 (0.131 g/100 g monacolin K). To eliminate the differences in food intake among groups, food intake was measured every day and adjusted. Following the 12-week period and an overnight fasting, all mice were weighed and euthanized under anesthesia, and tissues were collected. The tissues collected included blood, liver, and epididymal fat, as a representative of visceral fat. All procedures complied with the current guidelines for the Care and Use of Laboratory Animals, and the protocol was approved by the Committee on Animal Experimentation at the University of Toyama.

### 2.3. Evaluation of Serum Lipid, Glucose, and Insulin Levels, and Liver Function

Serum total cholesterol, LDL cholesterol, HDL cholesterol, and triglyceride (TG) levels were determined at LipoSearch (Skylight Biotech Inc., Akita, Japan). Serum-free fatty acid levels were measured by Determiner NEFA755 (Kyowa Medex Co., Ltd., Tokyo). Serum glucose levels were measured using the Wako Glucose CII-test (Wako Pure Chemical Industries, Osaka, Japan), and serum insulin levels were measured using the Ultrasensitive Insulin ELISA Kit (Morinaga Institute of Biological Science, Inc., Yokohama, Japan). Serum aspartate aminotransferase (AST) and alanine aminotransferase (ALT) levels were measured using the Wako Transaminase CII-test (Wako Pure Chemical Industries).

### 2.4. Serum Adipocytokine Levels

Adipocytokines (i.e., leptin, adiponectin) play an important role in the liver, peripheral glucose, and lipid metabolism as well as liver fibrosis and have been suggested as critical factors for NASH development. Serum leptin and adiponectin levels were measured using commercially available kits (mouse leptin ELISA kit, Shibayagi Co., Ltd., Gunma, Japan; adiponectin ELISA kit, Otsuka Pharmaceutical Co., Ltd., Tokyo).

### 2.5. Liver and Adipose Tissue Histopathology

Staining of frozen liver specimens with oil red O was performed at the Biopathology Institute Co., Ltd. (Oita, Japan). Formalin-fixed, paraffin-embedded liver and adipose tissues were processed, and 4 *μ*m thick serial sections were cut and stained with hematoxylin and eosin (HE) for histological examination. For immunohistochemistry, liver sections were incubated with rabbit anti-mouse TNF-*α* antibody (Monosan, Uden, The Netherlands), goat anti-mouse IL-6 antibody (R&D Systems, MN), rabbit anti-mouse CYP2E1 antibody (Enzo Life Sciences Inc., Farmingdale, NY), rabbit anti-mouse SMA antibody (Thermo Fisher Scientific, Cheshire, UK), and rabbit anti-mouse CD31 antibody (Thermo Fisher Scientific). We performed TUNEL staining using the ApopTag Plus Peroxidase In Situ Apoptosis Detection Kit, (Millipore, Billerica, MA) to detect apoptotic hepatocytes. Liver histology was scored using the system proposed by the NASH Clinical Research Network [11] based on four semiquantitative items, that is, steatosis (0–3), lobular inflammation (0–3), hepatocellular ballooning (0–2), and fibrosis (0–4). Three representative areas were scored in each section, and average values were used for the scoring. The sum of steatosis, lobular inflammation, and hepatocellular ballooning scores constituted NAS (NAFLD activity score) (Table 2). An NAS value equal or greater than five was consistent with a diagnosis of NASH while NAS < 3 allowed to rule out the diagnosis. Macrophages have been shown to aggregate, forming crown-like structures (CLSs) surrounding the necrotic adipocytes [12]. The number of adipocytes and CLSs in three fields at a magnification of 100x was determined by two independent pathologists blinded to each mouse group. 

### 2.6. Statistical Analysis

All continuous variables are expressed as the mean ± standard error of the measurement (SEM) and compared among groups using a one-way analysis of variance followed by Bonferroni correction for multiple testing. All analyses were two-tailed, and *P* < 0.05 was considered statistically significant. Statistical analysis was performed using StatView version 5.0 (Abacus Concept, Berkeley, CA, USA). 

## 3. Results

### 3.1. Body, Liver, and Visceral Fat Weight and Ratios

No significant differences were observed in body and organ weights at 24 weeks of age among all groups (Table 3). 

### 3.2. Serum Lipid Levels and Liver Function

No significant differences were observed in serum total cholesterol, LDL cholesterol, NEFA, and transaminase levels among all groups. Serum TG levels in the RYR-C group were significantly higher than those in the C and RYR-M groups (Table 3). 

### 3.3. Serum Glucose, Insulin, and Adipocytokine Levels

**Figure 1:**
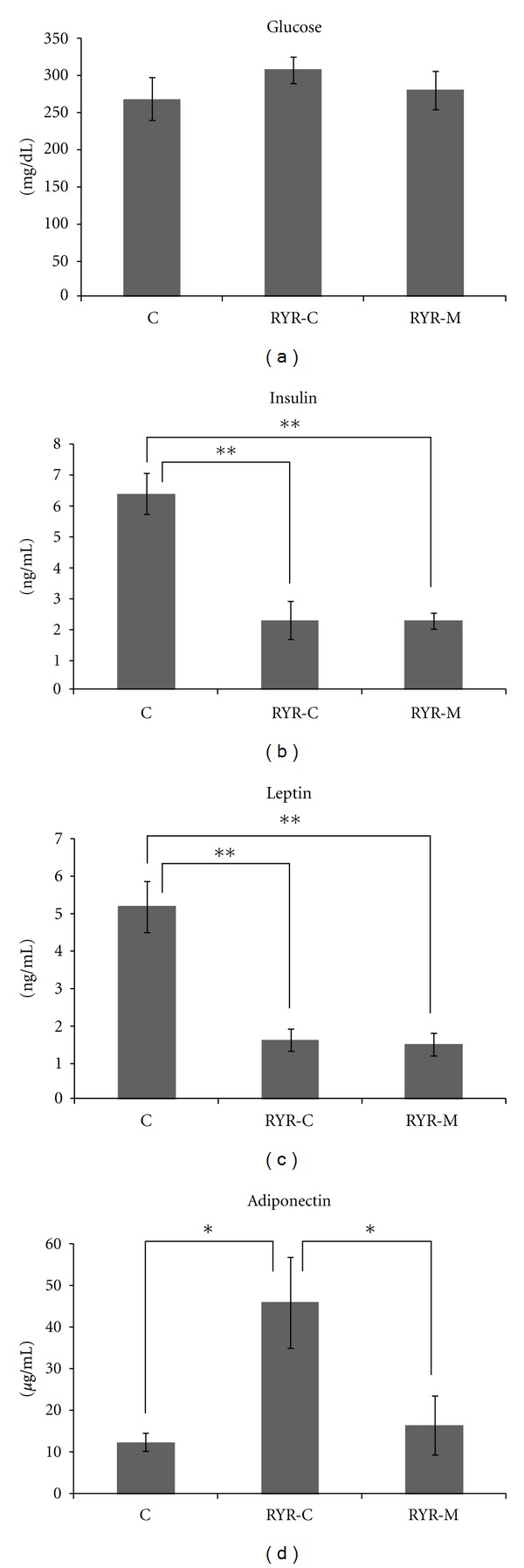
Glucose (a), insulin (b), leptin (c), and adiponectin (d) levels at 24 weeks of age in all experimental groups. All mice were administered MSG at birth. Variables are expressed as mean ± SEM. *P < 0.05; **P < 0.01.

Although no significant differences were observed in serum glucose levels among all groups, serum insulin levels in the RYR-M and RYR-C groups were significantly lower than those in the C group. Improvement in insulin resistance was observed in the RYR-M and RYR-C groups (Figure 1). Serum leptin levels in the RYR-M and RYR-C groups were significantly lower than those in the C group (Figure 1). Serum adiponectin levels in the RYR-C group were significantly higher than those in the C and RYR-M groups (Figure 1).

### 3.4. Histopathology

**Figure 2:**
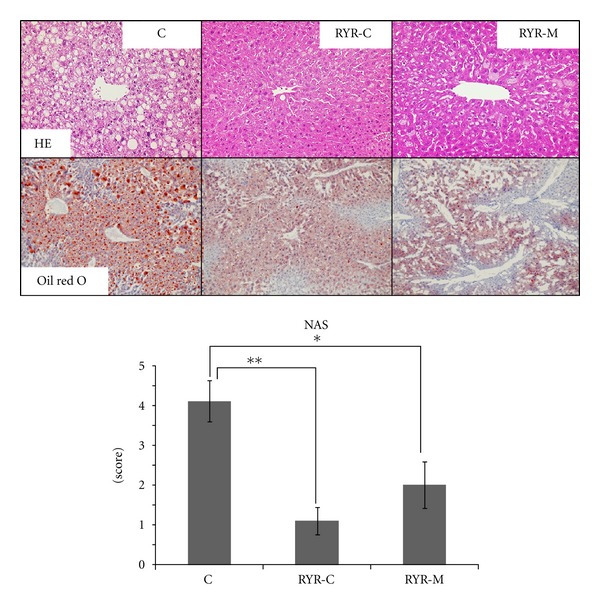
HE and oil red O staining of the liver tissue in all experimental groups. NAS was calculated after HE staining (100x) at 24 weeks in all experimental groups. All mice were administered MSG at birth. Representative tissue staining images are illustrated. Variables are expressed as mean ± SEM. Representative tissue staining images are illustrated. *P < 0.05; **P < 0.01.

**Figure 3:**
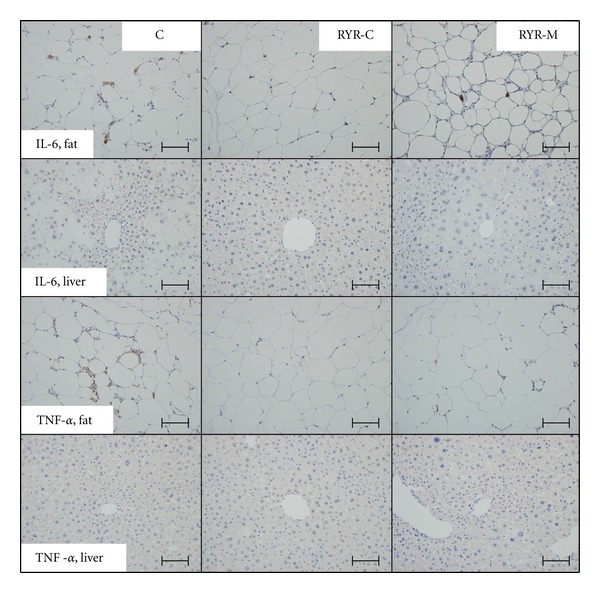
IL-6 and TNF-*α* immunohistochemical staining of the liver tissue and visceral fat at 24 weeks of age in all experimental groups. All mice were administered MSG at birth. Representative tissue staining images are illustrated. Scale bar means 100 *μ*m.

**Figure 4:**
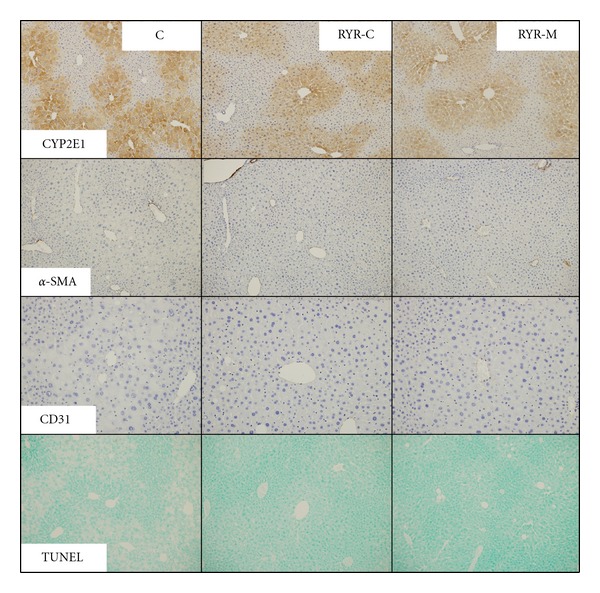
CYP2E1, *α*-SMA, and CD31 immunohistochemical staining and TUNEL staining of the liver tissue at 24 weeks of age in all experimental groups. All mice were administered MSG at birth. Representative tissue staining images are illustrated.

Oil red O staining revealed a large number of lipid droplets in the C group than in the RYR-M and RYR-C groups, who accumulated less lipids (Figure 2). IL-6 and TNF-*α* immunopositivity was not observed in the adipose tissue and liver of the RYR-C group. The RYR-M group contained IL-6 and TNF-*α* immunopositive cells scatteredly while the C group demonstrated a marked increase in immunopositivity in CLS macrophages of the adipose tissue (Figure 3). Furthermore CYP2E1 immunopositivity in the C group was higher than that in the RYR-M and RYR-C groups (Figure 4). Staining with the anti-*α*-SMA antibody revealed no fibrosis in the vascular smooth muscle cells of all the experimental groups (Figure 4). The endocapillary cells of the central vein showed CD-31 immunopositivity, but neovascularization was not observed (Figure 4). Finally, apoptotic hepatocytes were rarely observed in the livers of all experimental groups (Figure 4). 

**Figure 5:**
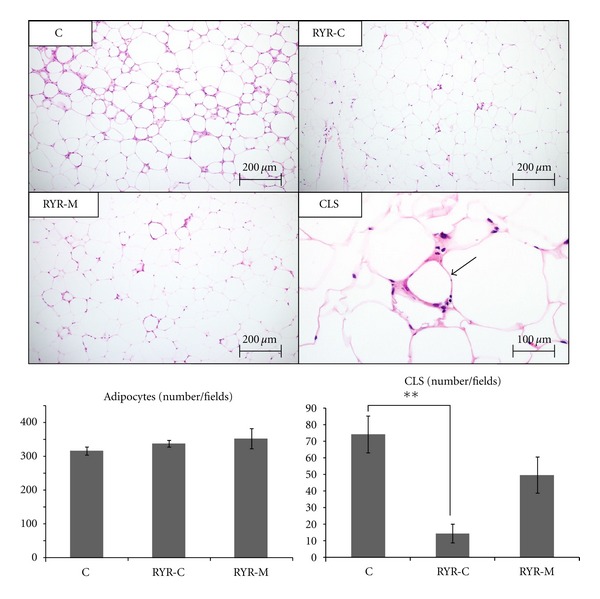
The number of adipocytes and CLSs was determined after HE staining of visceral fat at 24 weeks in the three experimental groups. All mice were administered MSG at birth. Representative tissue staining images are illustrated. CLS is illustrated by arrow. Representative tissue staining images are illustrated. Variables are expressed as mean ± SEM. **P < 0.01.

The NAS in the RYR-C and RYR-M groups was significantly lower than that in group C (Figure 2, Table 4). No significant differences in the number of adipocytes per unit area were observed among all the groups (Figure 5). The number of CLSs per unit area in the RYR-C group was significantly lower than that in the C group. In addition, although the number of CLSs per unit area was lower in the RYR-M group than that in group C, it was not statistically significant (Figure 5).

## 4. Discussion

This is the first study to compare the effects of RYR with different monacolin K levels prepared by the same manufacturer. We observed NAS in both RYR groups to be significantly lower than that in the C group. This finding suggests that RYR has inhibited NAFLD progression in a monacolin K level-independent manner. 

We found that both types of RYR (1P-DU and 3P-D1) improved insulin resistance and significantly decreased serum leptin levels without significant changes in weight compared to the control. In addition, we found RYR-C that contained low monacolin K levels significantly increased serum adiponectin levels compared to RYR-M that contains high monacolin K levels. Adiponectin inhibits fat accumulation in hepatocytes and inhibits progression to NASH by controlling the activation of hepatic stellate cells through AMPK [13, 14]. Although the number of adipocytes per unit area of the viewing field was not significantly different among all experimental groups, the number of CLSs in the RYR-C group was significantly lower than that in the C group.

CLSs represent the appearance of macrophages in clumps around the adipose tissue undergoing fatty necrosis [12]. We hypothesized that RYR may prevent necrosis by acting on the adipocytes through unknown mechanisms. Since there was no significant difference in the number of CLSs in visceral fat between the RYR-C and RYR-M groups, the degree of liver inflammation observed with RYR-M is probably because of enhanced inflammatory cytokines, such as TNF-*α* and IL-6 secreted by the adipocytes. Although IL-6 and TNF-*α*-immunopositivity was not detected in mouse liver immunohistochemistry samples, it was observed in the livers of patients with NAFLD [15, 16]. In CLSs of the mouse adipose tissue samples, IL-6 and TNF-*α*-immunopositivity was not detected in RYR-C group but was moderately presented in RYR-M group. An integrated response theory has been recently suggested in a NAFLD pathogenesis model instead of the two-hit theory [17, 18]. Taken together, our results suggested that IL-6 and TNF-*α* are mainly secreted by the adipocytes in visceral fat.

Liver cell apoptosis has been suggested as the second “hit” in the transition from simple NAFLD to NASH [19]. Considering this suggestion, our study found minor apoptosis in the MSG-administered mouse model of NAFLD, possibly because of incomplete NASH development at 24 weeks. Previous studies have correlated liver damage in NASH or in alcoholic liver disease with increased CYP2E1 isoenzyme activity [20] or expression [21], thus, suggesting the important role of CYP2E1 in NASH pathogenesis [22] through modulation of fatty acids [23]. Alpha-SMA antibody staining did not reveal fibrosis nor did CD31 antibody staining reveal neovascularization; however, as previously reported, MSG-administered mice normally develop liver fibrosis and hepatocellular carcinoma by 48 weeks of age, and not 24 weeks [10].

The PPAR-*γ* agonist pioglitazone has shown potential as a therapeutic drug for NASH [24]. Activation of PPAR-*γ* causes adipocyte differentiation and lipogenesis [25, 26]. Although serum adiponectin levels were significantly higher and serum leptin levels were significantly lower in the RYR-C group than in the C group, no significant differences in the number of adipocytes per unit area of viewing field and visceral fat to body weight ratio were observed among all experimental groups. RYR has been shown to inhibit PPAR-*γ* and leptin at the protein level in 3T3-L1 cells [27]; however, in this study, RYR did not act as a PPAR-*γ* agonist. Although insulin resistance improved significantly in the RYR-C and RYR-M groups compared with that in the C group, GABA contained in RYR may be one of the candidates to be the active constituent [28, 29].

We found no significant differences in serum total cholesterol, LDL cholesterol, and NEFA levels among all experimental groups, despite being treated with the statin monacolin K. The reason that no significant difference was observed in serum cholesterol levels has not been revealed in this study. Adjusted food consumption among three groups might act on serum cholesterol values. Lovastatin did not present serum cholesterol level lowering effect on the rodent model without hypercholesterolemia [30]. Monacolin K might not decrease the serum cholesterol value in status without hypercholesterolemia. Although many reports suggested that statins present lipid lowering effects, the rodent models take statins over 20 mg/kg/day [31–33]. In this study, mice took monacolin K only about 1 mg/kg/day in RYR-M. Intake of monacolin K might be too low to have an effect on the serum cholesterol value. Several clinical studies showed that statins are safe in patients with various chronic liver diseases including NAFLD and NASH [34–36]; therefore the clinical guideline recommends to prescribe statins for NAFLD and NASH [37]. Many studies have discussed RYR with a focus on monacolin K; however, monacolin is only one of its ingredients [38, 39]. There are few studies that have reported the efficacy of monacolin K in the treatment of NASH [40]. Our study suggests that not only monacolin K but also other ingredients of RYR may improve NASH. Furthermore, RYR is known to contain multiple medicinal properties [41]. In addition, a study reported that a two-month treatment with Japanese herbal medicines decreased total serum cholesterol levels to near standard values, although no change was evident when treatment was administered to patients who had an initial standard level of total serum cholesterol [42]. The NAFLD mouse model used in this study does not present high serum lipid levels at 24 weeks of age, which was the time of evaluation in this study. The reason why serum triglyceride values increased in RYR-C group has not been clarified in this study. The serum triglyceride value of RYR-C group was inadequate to provide a diagnostic criterion of hypertriglyceridemia. Ingredients of RYR except monacolin K may increase serum triglyceride level. In order to accurately assess if a component of RYR antagonizes monacolin K, it is necessary to evaluate another animal model that presents with high serum lipid levels over 24 weeks of age.

RYR is effective in treating dyslipidemia without the use of statins; however, the issue of varying medicinal properties due to nonstandard manufacturing methods is a problem [38]. Although the process of red yeast rice is described in the Bencao Gangmu, it does not mean that the process and the culture conditions are standardized among modern RYR manufacturers [43]. 

The two types of RYR used in this study significantly improved insulin resistance and decreased serum leptin levels. It is known that leptin upregulates the expression of procollagen-I, TGF-*β*1, and *α*-SMA [44]. RYR-C, with low monacolin K levels, significantly increased serum adiponectin levels compared with RYR-M, with high monacolin K levels. Moreover, only RYR-C significantly decreased the number of CLSs per unit area of viewing field compared with C group. Taken together, these findings suggest the pharmacological effect of RYR may vary with different components of RYR, which in turn varies according to the manufacturing process.

## 5. Conclusion

In summary, our findings indicate that the effects of RYR on NAFLD are independent of monacolin K levels and may be because of other RYR constituents, such as GABA. Although further study into how RYR decreases NAFLD progression is required, RYR should be considered as a therapeutic food source for NAFLD.

## Figures and Tables

**Table 1:** Chemical analysis of 1P-DU and 3P-D1.

Component	1P-DU g/100 g	3P-D1 g/100 g
Moisture	8.7	8.7
Crude protein	7.4	9.5
Crude fat	2.2	2.8
Crude ash	0.4	1.2
Crude carbohydrate	75.2	64.7
Crude fiber	6.1	13.1
Na	0.0077	0.0081
Monacolin K	0.002	0.131
GABA	0.032	0.069
Other amino acids	6.18	7.45

**Table 2:** NASH clinical research network scoring system.

	Item	Definition	Score/code
		Low-to-medium power evaluation of	
		parenchymal involvement by steatosis	
	Steatosis	<5%	0
		5–33%	1
		>33–66%	2
		>66%	3
NAS		Overall assessment of all inflammatory foci	
		No foci	0
	Lobular inflammation	<2 foci per 200 × field	1
		2–4 foci per 200 × field	2
		>4 foci per 200 × field	3
		None	0
	Hepatocellular ballooning	Few balloon cells	1
		Many cells/prominent ballooning	2
			Full score: 8

		None	0
		Perisinusoidal or periportal	1
Fibrosis	Stage	Mild, zone 3, perisinusoidal	1A
		Moderate, zone 3, perisinusoidal	1B
		Portal/periportal	1C
		Perisinusoidal and portal/periportal	2
		Bridging fibrosis	3
		Cirrhosis	4

**Table 3:** Body weight, organ weight, and serum parameters at 24 weeks of age in all experimental groups. All mice were administered MSG at birth. Variables are expressed as mean ± SEM.

Parameters	C	RYR-C	RYR-M
Body weight (g)	56.3 ± 1.1	51.8 ± 1.6	53.0 ± 2.0
Liver to body weight ratio (%)	4.8 ± 0.4	3.9 ± 0.3	4.1 ± 0.2
Visceral fat to body weight ratio (%)	2.9 ± 0.5	3.6 ± 0.3	3.0 ± 0.2
Total cholesterol (mg/dL)	137.1 ± 11.8	107.3 ± 7.8	116.5 ± 16.4
LDL cholesterol (mg/dL)	22.3 ± 3.5	16.2 ± 2.7	22.1 ± 5.3
TG (mg/dL)	48.2 ± 4.5	80.2 ± 5.1**	58.1 ± 5.3^†^
NEFA (mEq/L)	1.1 ± 0.1	1.0 ± 0.1	1.1 ± 0.1
AST (IU/L)	53.4 ± 7.0	41.4 ± 8.0	53.7 ± 9.4
ALT (IU/L)	28.1 ± 6.6	20.1 ± 6.1	29.8 ± 5.8

**Versus C *P* < 0.01, ^†^versus RYR-C *P* < 0.05.

**Table 4:** Score assessment of steatosis, lobular inflammation, hepatocellular ballooning, and NAS in C, RYR-C, and RYR-M.

Score			Steatosis (0–3)			Lobular inflammation (0–3)			Hepatocellular ballooning (0–2)		NAS (0–8)		
			0	1	2-3	0	1	2-3	0	1-2	0–2	3-4	5–8
C	(*n* = 9)	*n*	1	1	7	2	6	1	0	9	1	5	3
		(%)	(11.1)	(11.1)	(77.8)	(22.2)	(66.7)	(11.1)	(0)	(100)	(11.1)	(55.6)	(33.3)
RYR-C	(*n* = 9)	*n*	6	2	1	9	0	0	3	6	8	1	0
		(%)	(66.7)	(22.2)	(11.1)	(100)	(0)	(0)	(33.3)	(66.7)	(88.9)	(11.1)	(0)
RYR-M	(*n* = 9)	*n*	3	3	3	7	2	0	3	6	5	3	1
		(%)	(33.3)	(33.3)	(33.3)	(77.8)	(22.2)	(0)	(33.3)	(66.7)	(55.6)	(33.3)	(11.1)
